# Mediation of the Association Between *APOE* ε4 Genotype, Cognition, and Dementia by Neuropathology Imaging Markers in the Rotterdam Study

**DOI:** 10.1212/WNL.0000000000213679

**Published:** 2025-05-09

**Authors:** Jacqueline J. Claus, Mathijs T. Rosbergen, Jeremy A. Labrecque, Meike W. Vernooij, Frank J. Wolters, Mohammad Arfan Ikram

**Affiliations:** 1 Department of Epidemiology, Erasmus MC University Medical Center, Rotterdam, the Netherlands; and; 2 Department of Radiology & Nuclear Medicine and Alzheimer Center Erasmus MC, Erasmus MC University Medical Center, Rotterdam, the Netherlands.

## Abstract

**Background and Objectives:**

Insight into *APOE*-related pathways is important to unravel pathophysiology and identify therapeutic targets against late-life cognitive decline. We aimed to estimate mediators of *APOE ε*4 on cognition and dementia through different disease markers on structural in vivo brain imaging.

**Methods:**

All participants from the population-based Rotterdam Study who underwent brain MRI between 2005 and 2009 were included. Cognition was assessed cross-sectionally during center visits, and participants were followed up for incident dementia until January 1, 2020. Imaging markers included hippocampal volume (HV), volume of white matter hyperintensities (WMHs), Alzheimer disease–specific regional cortical thickness, and presence of ≥2 cerebral microbleeds. We performed causal mediation analyses to decompose the total effect of *APOE ε*4 carriership on cognition and dementia into natural direct and indirect effects and corresponding percentage mediated. We adjusted models for potential confounders.

**Results:**

Among 5,510 participants (mean age at time of MRI scan: 65.0 [±10.9] years, 55.0% women), 349 developed dementia, of whom 148 were ε4 carriers. Carriers of ε4 had slightly lower Z-scores for global cognition (β = −0.02 [-0.07 to 0.02], age-related cognitive decline = 4.4 months), with 7% (β = −0.00 [0.00–0.00]) of this association mediated by HV and 4% (β = −0.00 [−0.01 to 0.00]) by cortical thickness. In total, an estimated 25% of the effect of ε4 on cognition was mediated by microbleeds (*p* value = 0.24, [β = −0.00 {−0.01 to 0.00}]) and 12% by WMHs (*p* value = 0.44, [β = −0.00 {−0.01 to 0.00}]). In multiple mediator analyses, WMHs and microbleeds together accounted for 27% of the mediated effect of *APOE* ε4 on cognition (*p* value = 0.48). Carriers of ε4 had higher risk of incident dementia (HR 2.35 [95% CI 2.06–2.65]). For dementia, there was little to no evidence of mediation by either HV (3%, *p* value = 0.09, OR = 1.01 [1.00–1.03]) or regional cortical thickness (0%, *p* value = 0.79, OR = 1.00 [0.99–1.02]). In total, 1% of the effect of ε4 on dementia was mediated by WMHs (*p* value 0.29, OR = 1.00 [1.00–1.02]) and 5% by microbleeds (*p* value = 0.06), OR = 1.03 (1.00–1.07). In multiple mediator analyses, all 4 imaging markers together explained 6% of the mediated effect on incident dementia (*p* value = 0.04).

**Discussion:**

In this population-based cohort study, we found that an estimated one-fourth of the effect of *APOE* ε4 on cognition is mediated by structural brain imaging markers, driven mainly by cerebral microbleeds. For dementia, mediation by these markers was limited.

## Introduction

The apolipoprotein (*APOE*) ε4 gene is the strongest genetic risk factor of late-onset dementia and particularly Alzheimer disease.^1,2^ Homozygous carriers of the *APOE* ε4 allele have an absolute risk of 50% by age of 85 years to develop dementia due to Alzheimer disease, compared with less than 10% in noncarriers.^3^ Insight into the potential pathways that mediate the effect of *APOE* on cognitive decline and dementia is important to unravel pathophysiology and to identify potential therapeutic targets.^4^

Previous studies have highlighted the role of Alzheimer disease neuropathology, such as amyloid-β accumulation and tauopathy, in mediating the impact of *APOE* ε4 on cognitive decline.^1,5-7^ These processes could lead to volumetric loss in hippocampal volume (HV) and decreased cortical thickness, which could both be considered indicative of Alzheimer disease pathology, although not exclusively.^8-10^ However, there is increasing evidence that *APOE* ε4 also influences alternative processes contributing to late-life cognitive decline and dementia.^11,12^ For instance, the ε4 allele is associated with cerebrovascular pathology, including an increased burden of white matter hyperintensities (WMHs). Moreover, the ε4 allele is linked to cerebral amyloid angiopathy, which often leads to an increased number of microbleeds shown on brain MRI.^13,14^ Exploring the pathways from *APOE* ε4 to cognitive decline or dementia mediated by these neuroimaging biomarkers can further clarify the pathophysiologic processes of *APOE* ε4.

Previous insights from mediation studies are primarily derived from postmortem studies.^6,12,15-17^ However, antemortem studies measure neuropathologies before the development of dementia, enabling the establishment of a temporal relationship essential for determining causality. One recent study among 4,527 community-dwelling participants in Iceland did use in vivo MRI and observed that 25% of the overall detrimental effect of *APOE* ε4 on cognition was mediated by WMHs and total brain volume combined.^18^ While this limited proportion mediated could point toward alternative pathways, it might also be an underestimation due to imprecise measurement of cognition, for example, by day-to-day variation in measurements or influence of comorbid conditions such as depressive symptoms or delirium. Although this limitation could be overcome using dementia as a more robust outcome measure, dementia remains a dichotomous variable and, therefore, less sensitive than cognition, which provides a continuous and potentially more sensitive outcome.

We aimed to investigate the extent to which the effect of *APOE* ε4 genotype on cognition and dementia is mediated by structural brain imaging markers, using data from the population-based Rotterdam Study.

## Methods

### Study Population

This study was embedded within the population-based Rotterdam Study, an ongoing population-based study of determinants and occurrence of disease in persons aged 45 years and older. The study comprises 17,931 individuals living in the Ommoord suburb of Rotterdam, the Netherlands. The design of the Rotterdam Study has been described in detail previously.^19^ In brief, participants are invited for interview and extensive in-person examination at a dedicated research center about once every 3–6 years. We included all participants with available first brain MRI scan from 2005 to 2009 (n = 5,862) and excluded participants with no information on *APOE* genotype (n = 211), without informed consent for dementia follow-up (n = 32), without assessment of cognitive status at study start (n = 35), and with dementia at time of the MRI scan (n = 74) (eFigure 1). For cognition analyses, we further excluded those with incomplete cognitive assessment (n = 948).

### Ethics Approval

The Rotterdam Study has been approved by the Medical Ethics Committee of the Erasmus MC and by the Ministry of Health, Welfare and Sport of the Netherlands, implementing the Population Studies Act: Rotterdam Study. All participants provided written informed consent to participate in the study and to obtain information from their treating physicians.^19^

### APOE Genotype

*APOE* genotype was determined using PCR on coded DNA samples or using biallelic TaqMan assays (TaqMan Gene Expression Assays; Thermo Fisher Scientific, Waltham, MA) (rs7412 and rs429358).^20^ *APOE* ε4 carriers were defined as carrying at least one ε4 allele (ε3/ε4, ε2/ε4, and ε4/ε4). We used *APOE* ε3/ε3, ε2/ε2, and ε2/ε3 as a reference group, collectively referring to them as noncarriers. We did not differentiate between ε4 homozygotes and heterozygotes because of the limited number of homozygous ε4 carriers in the study (N = 124/5,510; 2.5%).

### Brain Imaging Markers

All participants underwent scanning on a 1.5T MRI scanner (GE Healthcare) using a multisequence protocol consisting of T1-weighted, proton density–weighted, fluid-attenuated inversion recovery (FLAIR), and T2-weighted sequences. For brain volumetry, T1-weighted (voxel size 0.49 × 0.49 × 1.6 mm^3^), proton density–weighted (voxel size 0.6 × 0.98 × 1.6 mm^3^), and the FLAIR (voxel size 0.78 × 1.12 × 2.5 mm^3^) scans were used for automated segmentation of supratentorial gray matter, white matter, CSF, and WMHs.^21,22^ All segmentations were visually inspected and manually corrected if needed. Supratentorial intracranial volume was estimated by summing gray and white matter (consisting of the sum of normal-appearing white matter and WMH volume) and CSF volumes.^22^ Furthermore, T1-weighted MR images were processed using FreeSurfer (version 6.0) to obtain HV^23^ and cortical thickness measurements. HV was defined as the sum of the left and the right HVs. We expressed HV, WMH volume, and intracranial volume as milliliters.

We used the mean cortical thickness in the surface area–weighted average of the Alzheimer disease cortical thickness regions,^24^ expressed in millimeters. This signature consists of multiple regions, including the entorhinal cortex, parahippocampus, inferior parietal lobe, pars opercularis, pars orbitalis, pars triangularis, inferior temporal, temporal pole, precuneus, supramarginal gyrus, superior parietal, and superior frontal regions.

All scans were appraised by trained research physicians for the presence of microbleeds and cortical and lacunar infarcts.^25^ We dichotomized microbleeds into those with or without ≥2 bleeds because previous community-based studies have shown associations of CMBs with cognitive function mostly in the presence of 2 or more microbleeds.^26,27^ Microbleeds in corticosubcortical and in the white matter lobar regions were considered lobar microbleeds.

### Cognitive Assessment

Assessment of global cognitive function was performed at center visits through a cognitive test battery, including the Stroop test,^28^ measuring processing and attention; the Word Fluency Test,^29^ measuring efficiency of searching long-term memory; the Letter Digit Substitution test,^30^ measuring processing speed and executive function; the 15-word verbal learning test (15-WLT),^31^ measuring verbal learning and memory; and the Purdue Pegboard test (PPB),^32^ measuring dexterity and fine motor speed. Cognition was assessed concurrently with brain MRI. We calculated global cognition (g-factor) from principal component analysis of these test scores and derived an additional g-factor that excluded PPB scores to remove the influence of motor function.^33^ The g-factor is defined as the first unrotated component of the principal component analysis. A higher g-factor indicates better cognitive performance.

### Dementia Diagnosis

Participants were assessed for dementia both at baseline and during follow-up evaluations conducted every 4–6 years^34^ Screening included the Mini-Mental State Examination (MMSE) and the Geriatric Mental Schedule (GMS) organic level.^34^ Individuals with a positive result (defined as MMSE score <26 or GMS score >0) underwent further assessment, which included a detailed examination and an informant interview using the Cambridge Examination for Mental Disorders in the Elderly.^34^ When necessary, additional neuropsychological tests were performed for those suspected of having dementia.^34^ The entire cohort was also monitored for dementia on an ongoing basis through a computerized system that linked the study database to electronic medical records from general practitioners, who play a central role in the Dutch health care system and coordinate all medical information for their patients, as well as to records from the Regional Institute for Outpatient Mental Health Care.^34^ A final diagnosis of dementia was made by a consensus panel led by a neurologist, based on standard diagnostic criteria, including the DSM-III-R for dementia and the NINCDS-ADRDA criteria for clinical Alzheimer disease.^34^

### Assessment of Covariables

Educational attainment was assessed at study entry and subdivided into 3 categories: primary, intermediate, and higher vocational education. Smoking habits were assessed by interview and categorized as never, former, or current smoking. Body mass index was calculated from measured weight and height (kg/m^2^). Blood pressure was measured twice using a random-zero sphygmomanometer, and we used the average of 2 readings. We included systolic blood pressure and non–high-density lipoprotein (total cholesterol minus high-density lipoprotein cholesterol) as continuous measures.^35^ We included self-reported use of antihypertensive medication and lipid-lowering drugs. Participants were continuously followed up for the occurrence of diabetes mellitus, coronary heart disease, and atrial fibrillation through a combination of in-person assessment and with electronic linkage of medical records. Ethnicity was self-reported using parental birth country.

### Data Analysis

We had complete assessment for covariables including age, sex, and stroke. Missing data for covariables including diabetes mellitus (1.0%), atrial fibrillation (1.0%), and antihypertensive medication (0.6%) were imputed using fivefold multiple imputations.

We transformed WMH volumes to allow for normality of the data using the natural logarithm (ln). We then standardized ln-transformed WMH volume, cortical thickness in the Alzheimer disease–specific region, and HV.

We estimated the effect of carrying the *APOE* ε4 allele on global cognition in terms of equivalent years of aging by first modeling the association between age and global cognition using linear regression and then dividing the β-coefficient for *APOE* ε4 by the β-coefficient for age.

We performed causal mediation analysis based on the counterfactual outcome framework to quantify the effect of *APOE* ε4 genotype on cognition and dementia that is mediated by each imaging marker (HV, cortical thickness, WMHs, and microbleeds). The counterfactual framework is an approach for estimating causal effects by comparing observed outcomes with hypothetical, or “counterfactual,” scenarios.^36^ We used a regression-based approach to calculate the total, direct, and indirect effects.^36^ We decomposed the total effect into 2 nonoverlapping components: (1) the natural indirect effect (the mediated effect, from exposure through a mediator to the outcome) and (2) the natural direct effect (the direct path from exposure to outcome, not through the mediator), while allowing for interaction between *APOE* genotype and the mediator. The indirect effect is the effect of *APOE* ε4 genotype on cognition or dementia acting through brain imaging markers. Furthermore, we calculated the proportion mediated by dividing the indirect effect by the total effect. For these effects to be reliable causal estimates, we adjusted for several potential confounders of the exposure-outcome association, mediator-outcome association, and exposure-mediator association. We, therefore, adjusted all models for age, sex, education, smoking status, body mass index, hypertension, antihypertensive medication, non–high-density lipoprotein cholesterol, lipid-lowering drugs, diabetes, history of stroke, and atrial fibrillation. Models including HV, cortical thickness, and WMHs further included intracranial volume. To derive 95% CIs of these estimates, we used bootstrapping with 1,000 replications.

We performed several sensitivity analyses, (1) stratifying on age older than 75 years for dementia analyses; (2) including only clinical Alzheimer disease dementia; (3) excluding participants with cortical infarction on brain MRI for analyses including WMH and cerebral microbleed mediators; (4) restricting on follow-up time for dementia analyses, that is, by censoring follow-up at 3, 5, 10, and 15 years and including only dementia cases that happened within each specified period; (5) stratifying on sex; and (6) using the cognition g-factor, excluding motor function (as assessed by the PPB).

Last, we performed multiple mediator analyses to estimate the joint effect of the neuroimaging markers on the proportion mediated for both cognition and dementia.^37^

All analyses were performed in R (version 4.2.1) using the packages “mice” and “CMAverse.”

## Results

Among 5,510 participants, 1,557 (28.2%) were *APOE* ε4 carriers. The mean age at the time of MRI scan was 65.0 (±10.9) years, and 3,032 (55.0%) were women (Table 1). *APOE* ε4 carriers had similar baseline characteristics compared with noncarriers. Neuroimaging markers were similar across *APOE* genotypes, although ε4 carriers exhibited a slightly higher prevalence of microbleeds with more lobar microbleeds, compared with noncarriers (Table 1). During a median 11-year follow-up, 349 of 5,510 participants (6.3%) developed dementia, of whom 148 (42.4%) were ε4 carriers. Follow-up time was similar between *APOE* ε4 carriers (median 10.6 years, interquartile range [IQR] 7.9–12.3) and noncarriers (10.5 years, IQR 8.1–12.5). Baseline characteristics were similar for the subsample of 4,562 participants with complete cognitive assessment (eTable 1).

**Table 1:** Demographic and Clinical Characteristics for *APOE* ε4 and Reference (ε3 and ε2) Carriers and the Whole Cohort

	Whole cohort	*APOE* ε2 and ε3 carriers^a^	*APOE* ε4 carriers^b^
	N = 5,510	N = 3,953 (71.7%)	N = 1,557 (28.3%)
Female sex	3,032 (55.0)	2,181 (55.2)	851 (54.7)
Age (y), mean (SD)	65.0 (10.9)	65.35 (11.03)	64.1 (10.5)
Education			
Primary	494 (9.0)	361 (9.2)	133 (8.6)
Lower/intermediate	2,096 (38.3)	1,525 (38.8)	571 (37.0)
Intermediate vocational	1,670 (30.5)	1,206 (30.7)	464 (30.1)
Higher vocational	1,214 (22.2)	838 (21.3)	376 (24.4)
Smoking			
Never	1,651 (30.0)	1,234 (31.3)	417 (26.9)
Current	2,727 (49.6)	1,907 (48.4)	820 (52.8)
Former	1,117 (20.3)	801 (20.3)	316 (20.3)
Body mass index, mean (SD)	27.5 (4.2)	27.59 (4.23)	27.2 (4.0)
Non–high-density lipoprotein cholesterol, mean (SD)	4.1 (1.0)	4.0 (1.0)	4.2 (1.1)
Lipid-lowering medication	1,385 (25.3)	978 (24.8)	407 (26.3)
Systolic blood pressure, mean (SD)	140.2 (21.6)	140.84 (21.90)	138.7 (20.7)
Blood pressure–lowering medication	2,009 (36.7)	1,472 (37.5)	537 (34.7)
Diabetes mellitus	705 (12.9)	531 (13.4)	174 (11.3)
Atrial fibrillation	262 (4.8)	204 (5.2)	58 (3.8)
History of stroke	181 (3.3)	126 (3.2)	55 (3.5)
Hippocampal volume milliliter, mean (SD)	7.8 (0.9)	7.7 (0.9)	7.8 (0.9)
Alzheimer disease–specific cortical thickness mm, mean (SD)	2.5 (0.1)	2.5 (0.1)	2.5 (0.1)
White matter hyperintensities ml, median (IQR)	3.1 (1.4)	3.1 (1.4)	3.1 (1.4)
Intracranial volume ml, mean (SD)	1,140.1 (116.1)	1,138.3 (116.9)	1,144.5 (114.2)
≥2 Microbleeds present	449 (8.2)	291 (7.4)	158 (10.2)
≥2 Lobar microbleeds present	343 (6.2)	220 (5.6)	113 (7.3)
Northwest European background	4,958 (93.7)	3,547 (93.5)	1,411 (94.3)

Abbreviations: *APOE* = apolipoprotein; ml = milliliter; mm = millimeter; IQR = interquartile range.

Numbers are n (%) unless otherwise specified.

a Includes *APOE* ε3/ε3, ε2/ε2, and ε2/ε3.

b Includes ε4/ε4, ε4/ε3, and ε4/ε2.

### Mediation by HV and Cortical Thickness

**Figure 1:**
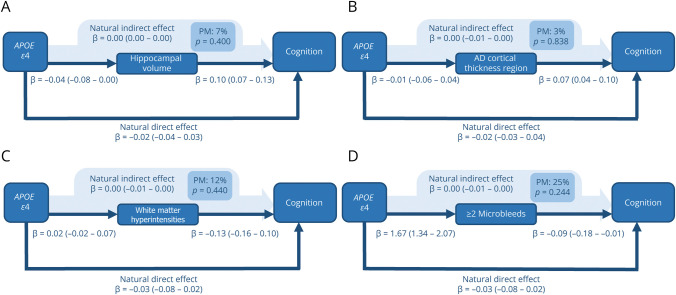
Mediation of *APOE* ε4 Effects of Neuroimaging Markers on Cognition These directed acyclic graphs incidate the natural direct effect; the natural indirect effect; and the percentage mediated by (A) hippocampal volume, (B) Alzheimer disease cortical thickness region, (C) white matter hyperintensities, and (D) the presence ≥2 of microbleeds. Each arrow indicates a causal relationship and points from the exposure toward the outcome computed in that particular model. The natural direct effect, the natural indirect effect, and the proportion mediated are computed using causal mediation analyses according to the counterfactual framework, with linear and logistic regression regression models. 95% CIs are indicated in parentheses. Reference groups for *APOE* ε4 are *APOE* ε3/ε3, ε2/ε3, and ε2/ε2 carriers. All models are adjusted for potential confounders (age, sex, education, smoking status, body mass index, non–high-density lipoprotein cholesterol, lipid-lowering drugs, systolic blood pressure, blood pressure–lowering drugs, diabetes mellitus, atrial fibrillation, stroke). Models including hippocampal volume, Alzheimer disease cortical thickness region, and white matter hyperintensities were further adjusted for intracranial volume. AD = Alzheimer disease; *APOE* = apolipoprotein E; OR = odds ratio; PM = proportion mediated; *p* = *p* value.

**Figure 2:**
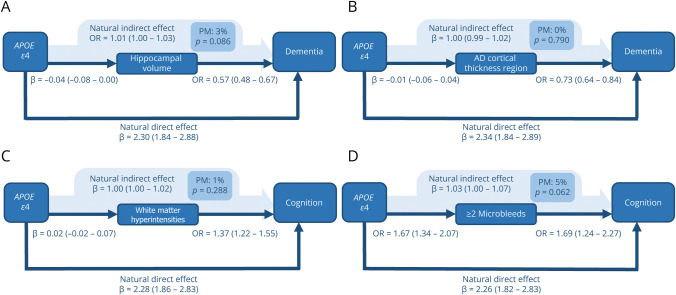
Mediation of *APOE* ε4 Effects of Neuroimaging Markers on Dementia These directed acyclic graphs incidate the natural direct effect; the natural indirect effect; and the percentage mediated by (A) hippocampal volume, (B) Alzheimer disease cortical thickness region, (C) white matter hyperintensities, and (D) the presence ≥2 of microbleeds. Each arrow indicates a causal relationship and points from the exposure toward the outcome computed in that particular model. The natural direct effect, the natural indirect effect, and the percentage mediated are computed using causal mediation analyses according to the counterfactual framework, with logistic and linear regression regression models. 95% CIs are indicated in parentheses. Reference groups for *APOE* ε4 are *APOE* ε3/ε3, ε2/ε3, and ε2/ε2 carriers. All models are adjusted for potential confounders (age, sex, education, smoking status, body mass index, non–high-density lipoprotein cholesterol, lipid-lowering drugs, systolic blood pressure, blood pressure–lowering drugs, diabetes mellitus, atrial fibrillation, stroke). Models including hippocampal volume, Alzheimer disease cortical thickness region, and white matter hyperintensities were further adjusted for intracranial volume. AD = Alzheimer disease; *APOE* = apolipoprotein E; OR = odds ratio; PM = proportion mediated.

*APOE* ε4 genotype was not significantly associated with lower HV or lower regional cortical thickness when compared with noncarriers (Figures 1 and 2). Carriers of ε4 had slightly lower Z-scores for cognition (β = −0.02 [95% CI −0.07 to 0.02]). This cognitive difference is equivalent to an age-related cognitive decline of approximately 4.4 months (95% CI −4.4 to 13.1). Of this effect, 7% (*p* value 0.400) was mediated by HV and 3% (*p* value 0.838) by cortical thickness in the Alzheimer disease–specific regions (Figure 1). Proportions mediated remained similar for cognitive Z-scores excluding the PPB scores (HV 5%, *p* value 0.572, cortical thickness 6%, *p* value 0.536). ε4 carriers had a higher risk of incident dementia (OR 2.35 [95% CI 2.06–2.65]), but there was little evidence of mediation by either HV (3%; *p* value 0.086) or Alzheimer disease–specific cortical thickness (0%; *p* value 0.790) (Figure 2). Mediation estimates remained similar in sensitivity analyses stratifying on age (older than 75 years, HV: 3%, *p* value 0.092; cortical thickness: 0%, *p* value 0.774), and on Alzheimer disease dementia only (HV: 2%, *p* value 0.088; cortical thickness: 0%, *p* value 0.696). When restricting the analyses by follow-up duration, the mediated effect of *APOE* ε4 on dementia was slightly higher at 7% (*p* value 0.134) for dementia within the first 3 years after the MRI scan date (eTable 2). In sex-stratified analyses, mediation effects by HV and cortical thickness were stronger in women than in men for cognition and to a lesser extent for dementia (eTable 3). However, owing to smaller sample sizes, these results should be interpreted with caution.

### Mediation by WMHs and Microbleeds

Carriers of the *APOE* ε4 allele tended to have a slightly higher volume of WMHs, although not statistically significant, and more often had ≥2 microbleeds than noncarriers (OR 1.61, 95% CI 1.29–2.01). This association was similar for ≥2 lobar microbleeds (OR 1.67, 95% CI 1.31–2.13). In total, 25% of the effect of ε4 on cognition was mediated by presence of ≥2 microbleeds, which was similar for the presence of ≥2 lobar microbleeds (23%, *p* value 0.230), and 12% was mediated by volume of WMHs (Figure 2). This proportion mediated was lower when excluding the PPB test from cognitive Z-scores, with 12% (*p* value 0.180) mediated by presence of ≥2 microbleeds and 9% (*p* value 0.344) by WMHs.

Mediation effects by WMHs on cognition were similar in men (31.4%) and women (30.0%) (eTable 3). For ≥2 microbleeds, mediation effects were larger in men than in women (10.2 vs 1.6%; eTable 3).

The share of effect mediation was less pronounced in the association between ε4 and dementia, of which only 1% was mediated by WMHs and 5% by ≥ 2 microbleeds, similar for ≥2 lobar microbleeds (3%, *p* value 0.110) (Figure 1). This remained similar in sensitivity analyses only including clinical Alzheimer disease dementia (WMHs 1%, *p* value 0.350, ≥2 microbleeds 5%, *p* value 0.060). In sensitivity analyses excluding participants with cortical infarction on MRI, the proportion mediated remained similar for WMHs at 11% (*p* value 0.566) for cognition and 1% (*p* value 0.458) for dementia, as well as for microbleeds (cognition: 23% *p* value 0.352; dementia 5% *p* value 0.008). Mediation effects of ≥2 microbleeds on dementia were larger in men (10.2%) than in women (1.6%) (eTable 3).

### Multiple Mediator Analysis

In multiple mediator analyses, HV and ≥2 microbleeds had the highest combined proportion mediated for both cognition (32%, *p* value 0.356) and dementia (7%, *p* value 0.004) (Table 2). WMHs and ≥2 microbleeds together accounted for 27% of the mediated effect of *APOE* ε4 on cognition and 5% on dementia. All 4 imaging markers together explained 31% of the mediated effect of *APOE* ε4 on cognition and 6% on incident dementia (Table 2).

**Table 2:** Mediation Effects of Multiple Neuroimaging Markers on the Association of *APOE* ε4 With Cognition and Dementia

Outcome: cognition					
	Total effect	Direct effect	Indirect effect	Proportion mediated (%)	*p* Value of mediated effect
	*APOE* ε4 → cognition	*APOE* ε4 → other pathways → cognition	*APOE* ε4→ mediator → cognition		
HV + WMH	−0.02 (−0.07 to 0.03)	−0.02 (−0.07 to 0.03)	0.00 (−0.01 to 0.00)	17	0.552
HV + MB	−0.02 (−0.08 to 0.02)	−0.02 (−0.07 to 0.03)	0.00 (−0.01 to 0.00)	32	0.356
WMH + MB	−0.02 (−0.08 to 0.02)	−0.02 (−0.07 to 0.03)	−0.01 (−0.02 to 0.00)	27	0.476
HV + WMH + MB	−0.02 (−0.07 to 0.02)	−0.02 (−0.07 to 0.03)	−0.01 (−0.02 to 0.00)	32	0.464
HV + MB + WMH + AD cortical thickness	−0.02 (−0.07 to 0.03)	−0.02 (−0.07 to 0.03)	−0.01 (−0.02 to 0.00)	31	0.442

Outcome: incident dementia					
	Total effect	Direct effect	Indirect effect	Proportion mediated (%)	*p* Value of mediated effect
	*APOE* ε4→ dementia	*APOE* ε4 → other pathways → dementia	*APOE* ε4 → mediator → dementia		
HV + WMH	2.37 (1.88–2.98)	2.32 (1.83–2.92)	1.02 (1.00–1.04)	3	0.078
HV + MB	2.35 (1.87–2.95)	2.26 (1.80–2.82)	1.04 (1.01–1.09)	7	0.004
WMH + MB	2.35 (2.06–2.65)	2.31 (2.00–2.64)	1.03 (1.00–1.09)	5	0.200
HV + WMH + MB	2.34 (1.86–2.62)	2.26 (1.84–2.57)	1.04 (1.01–1.06)	6	<0.001
HV + MB + WMH + AD cortical thickness	2.34 (1.87–2.92)	2.26 (1.80–2.79)	1.03 (1.00–1.09)	6	0.036

Abbreviations: AD cortical thickness = cortical thickness in Alzheimer disease–specific region; *APOE* = apolipoprotein; HV = hippocampal volume; MB = microbleed; WMH = white matter hyperintensity.

For cognition, values for total, direct, and indirect effects indicate changes in Z-scores for global cognition. For dementia, values for total, direct, and indirect effects indicate odds ratios for incident dementia. Values in between parentheses indicate 95% CIs. Effect estimates are adjusted for age, sex, education, smoking status, body mass index, non–high-density lipoprotein cholesterol, lipid-lowering drugs, systolic blood pressure, blood pressure–lowering drugs, diabetes mellitus, atrial fibrillation, stroke, and intracranial volume.

## Discussion

In this population-based cohort study, we found that approximately one-fourth of the effect of *APOE* ε4 on cognition is mediated by structural brain imaging markers, mainly driven by the presence of cerebral microbleeds. Notably, neuroimaging markers of Alzheimer disease, such as HV and cortical thickness in Alzheimer disease–specific regions, showed minimal mediation effects on both cognition and dementia. All 4 neuroimaging markers combined mediated only 6% of the effect of *APOE* ε4 on dementia, suggesting either that cognition as an outcome better captures the mediated variation than dementia or that cognition is more profoundly affected by the studied mediators.

Our findings emphasize the substantial role of cerebrovascular pathology in the development of cognitive decline in middle-aged to older (mean age 65 years) carriers of the *APOE* ε4 allele. Most evidence thus far originated from postmortem studies, including older individuals, suggesting little to no mediation of the effect of *APOE* ε4 on late-life cognition by markers of vascular disease on brain autopsy.^7,15,16^ The measures of vascular disease in these studies included atherosclerosis, lacunar infarcts, microinfarcts, macroinfarcts, and hemorrhages. Antemortem studies capture neuropathology before dementia development and, therefore, can establish a temporal relationship, which is essential to establish causality. One previous antemortem mediation study investigated the effect of *APOE* ε4 on cognition using brain MRI in community-dwelling older individuals in Iceland and found 9% mediation by WMHs.^18^ Taken together with our findings, this suggests that the effects of *APOE* ε4 on cognition are for an important part mediated by cerebral small vessel disease. By contrast, mediation by brain volume seems more limited, with 6% mediation by HV in this study and 15% mediation by total brain volume in the Icelandic cohort.^18^ Total brain volume likely reflects the common end point of multiple pathologies, which is why we focused on more specific, intermediary markers such as HV in this study. Although the hippocampus itself can be affected by Alzheimer disease as well as limbic-predominant age-related TDP-43 encephalopathy (LATE) and vascular pathology, it might provide some more specific information about Alzheimer disease.^38,39^ Similarly, using regional cortical thickness, we attempted to better capture the Alzheimer component of neurodegeneration, but mediation by atrophy of either the hippocampus or Alzheimer-specific brain regions was limited.^40^

It is important to note that the associations between *APOE* ε4 and cognition were relatively weak, possibly due to the relatively young age of our study population. Therefore, the contribution of vascular effects to cognition should be interpreted as having only a modest influence. Because the effect of *APOE* ε4 on cognition is stronger in older populations, future research could examine whether mediation effects remain consistent in an older cohort.

Cerebral microbleeds accounted for most of the mediation in our analyses. The etiology of vascular disease in the brain leading to microbleeds can vary. Hypertension and atherosclerosis primarily cause deep microbleeds while cerebral amyloid angiopathy is associated with lobar microbleeds.^41-43^ Our exploratory, secondary analyses suggest that mediation was primarily due to lobar microbleeds. This suggests that the effect of *APOE* ε4 on cognition for a substantial part was due to cerebral amyloid angiopathy. However, the diagnostic value of neuroimaging criteria for cerebral amyloid angiopathy has not been validated in the general population, and more specific markers of cerebral amyloid angiopathy are needed to confirm or refute this suggestion.^44-46^

While close to one-third of *APOE* effects on cognition could be explained by neuroimaging markers jointly, this also leaves two-thirds incomplete captured by these markers or due to alternative pathways. It is important to note that mediation effects on the association between *APOE* ε4 and incident dementia were considerably smaller for dementia than for cognition. This discrepancy between cognition and dementia is challenging to explain biologically because the current scientific paradigm dictates a continuum from gradual loss of cognitive ability to the onset of dementia. We consider several potential explanations for these paradoxical findings. First, in our study, the effects of *APOE* ε4 on cognition were small, but a large proportion of these effects were driven by vascular factors. By contrast, the effect of *APOE ε4* on dementia was stronger, yet vascular effects played a less mediating role. A possible, albeit somewhat speculative, biological explanation is that cerebrovascular pathology due to *APOE* ε4 leads to cognitive decline, but additional “hits” may be required for the transition to dementia, involving a more complex interplay between pathologies.^47^ Second, a continuous Z-score for cognitive assessment may be more sensitive in capturing the effects of *APOE* and its mediators compared with a binary dementia diagnosis, which lacks the granularity needed to capture more subtle preclinical cognitive decline.Third, certain differences in the modeling approach between the cognition and dementia analyses may have contributed to a variation in mediation effects. Notably, cognition was assessed at time of MRI while dementia incidence occurred up to 15 years later. Nevertheless, various sensitivity analyses aimed at addressing these differences provided similar results. Future studies are needed to replicate our findings and explore alternative pathophysiologic pathways to incident dementia in a causal mediation framework.

Our findings could have implications for drug development against late-life cognitive decline and dementia. Most of the drug trials in Alzheimer disease dementia are targeted at the removal of amyloid-β, showing large decreases in amyloid-β on PET scans, but only modest to no effects on cognitive impairment and clinical outcomes.^48^ We found that over one-fourth of the effect of *APOE* ε4 on cognition is mediated by cerebrovascular factors, suggesting that the ε4 allele exerts its effect on late-life cognitive impairment not solely through the accumulation of β-amyloid plaques. Indeed, 1 previous mediation study showed that the mediated effects of *APOE* on cognition by non–Alzheimer disease neuropathologies were consistent, independent of presence of β-amyloid.^12^

Although we aimed to capture Alzheimer pathology using proxies such as HV and cortical thickness, measures of β-amyloid or tau were not available in this study. Future research on the effects of *APOE* would benefit from incorporating both amyloid and tau pathology assessments, either through imaging or plasma, in addition to cerebrovascular imaging. It is important to acknowledge diagnostic limitations of each of these methods for detecting underlying pathology. For instance, specificity of tau-PET may be limited while MRI criteria for cerebral amyloid angiopathy have not been validated in community-based samples.^49,50^

This study is strengthened by its large, population representative sample of individuals with in vivo brain imaging, cognitive assessment, and long-term follow-up for dementia. When interpreting our findings, some limitations also need to be acknowledged. First, there may have been uncontrolled confounding factors influencing the relationship between the mediator and the outcome variables. Consequently, the proportion mediated could be overestimated in the presence of these unmeasured confounders. Second, we had no biomarker-confirmed diagnosis of dementia etiology in participants who developed dementia during follow-up, and the importance of various *APOE*-related pathways may differ between dementia subtypes. Nevertheless, results provide relevant information on the importance of the investigated pathways for overall risk reduction strategies. Third, the number of *APOE* ε2 and homozygous ε4 carriers was small, limiting analyses specific to these subgroups. Future joint research efforts could combine data from multiple population-based cohorts to increase the sample size of *APOE* ε2 and homozygous ε4 carriers. Last, it is important to acknowledge that our study population was predominantly White, potentially limiting the generalizability to non-White populations, in whom effects of *APOE* ε4 on cognitive decline may be less prominent.

In conclusion, we found that one-fourth of the effect of *APOE* ε4 on cognition was mediated by structural neuroimaging markers, mainly driven by the presence of cerebral microbleeds. For dementia, mediation by these markers was limited.

## Glossary

FLAIR: fluid-attenuated inversion recovery
GMS: Geriatric Mental Schedule
HV: hippocampal volume
IQR: interquartile range
MMSE: Mini-Mental State Examination
PPB: Purdue Pegboard test
WMH: white matter hyperintensity
